# Biomolecular Strategies for Vascular Bundle Development to Improve Crop Yield

**DOI:** 10.3390/biom12121772

**Published:** 2022-11-28

**Authors:** Wei Chang, Hongqiao Chen, Guixiang Jiao, Yi Dou, Lin Liu, Cunmin Qu, Jiana Li, Kun Lu

**Affiliations:** 1 Integrative Science Center of Germplasm Creation in Western China (Chongqing) Science City and Southwest University, College of Agronomy and Biotechnology, Southwest University, Chongqing 400715, China; 2 Engineering Research Center of South Upland Agriculture, Ministry of Education, Chongqing 400715, China; 3 Academy of Agricultural Sciences, Southwest University, Chongqing 400715, China

**Keywords:** vascular bundle, crop yield, biomolecular strategies, source-flow-sink, ideal plant architecture, external environment

## Abstract

The need to produce crops with higher yields is critical due to a growing global population, depletion of agricultural land, and severe climate change. Compared with the “source” and “sink” transport systems that have been studied a lot, the development and utilization of vascular bundles (conducting vessels in plants) are increasingly important. Due to the complexity of the vascular system, its structure, and its delicate and deep position in the plant body, the current research on model plants remains basic knowledge and has not been repeated for crops and applied to field production. In this review, we aim to summarize the current knowledge regarding biomolecular strategies of vascular bundles in transport systems (source-flow-sink), allocation, helping crop architecture establishment, and influence of the external environment. It is expected to help understand how to use sophisticated and advancing genetic engineering technology to improve the vascular system of crops to increase yield.

## 1. Introduction

Crop yields have increased as a result of technological advances, surpassing the negative effects of current environmental conditions on crop development and yield in recent decades [1], such as climate change, flooding, drought, salinity, pests and pathogens, and other multiple co-occurring environmental stress factors [2,3,4,5,6]. Reductions in available farmland and the ever-increasing global population also lead to worldwide food shortages. One of the sustainable development goals proposed by the United Nations is to achieve zero hunger by 2030 and beyond [7], but based on current crop yield trends, it will still be insufficient to feed the world’s population by 2050 [1]. Moving forward, in order to meet the growing demand for stable crop yields while working with diminishing resources, innovative strategies and comprehensive analysis will be necessary.

From an evolutionary point of view, vasculature is crucial for life, whether in vertebrates [8] or plants [9,10,11,12]. In plants, the vascular system runs through the whole plant body, especially the leaves, stem, and roots, and plays an important role in nutrient and inorganic substance transport, mechanical support, and strength [8,9]. The vascular system has been relatively less studied compared to other plant systems, despite its invaluable importance. Vascular tissues are challenging to study experimentally because they are firmly embedded throughout the plant body [13]. The vascular system is complex, each vascular bundle has three distinct cell types (procambium/cambial, xylem, and phloem) with specialized functions arranged in a highly organized manner [8]. On this basis, it is widely recognized that xylem and phloem cells are commonly derived from procambium/cambial cells [14], and they are involved in the processes of long-term development and continuous differentiation, which have complex regulatory networks and undoubtedly increase the difficulty of its research.

**Figure 1:**
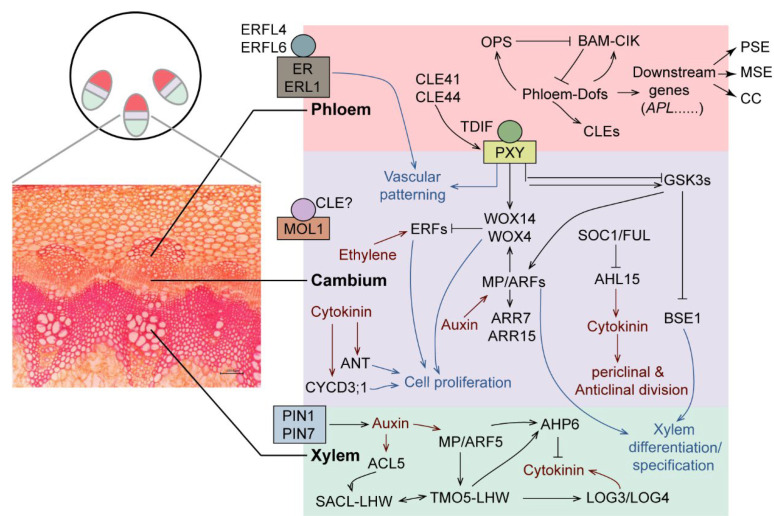
Complex genetic networks involving multiple transcription factors and phytohormones underlying vascular tissue specification. Individual vascular bundles composed of procambial cells between xylem (inner) and phloem (outer) in dicot angiosperms. Phloem formation, Phloem-Dofs activate the production of secretory CLE peptides, which are perceived by the BAM-CIK receptor system, reduce phloem-Dof proteins and repress formation of phloem cells. Phloem-Dofs also induce OPS, which is a negative regulator of BAM-CIK. The phloem-Dof positive feedback loop and the OPS-mediated BAM-CIK inhibition counteract the effect of CLE in SE- and CC-committed cells. Cambial specification PXY is activated by its ligands, CLE41 and CLE44. TDIF peptide as CLE family member is produced in the phloem and presumably moves to the cambium, where it binds to its receptor PXY and upregulates the expression of WOX4 and WOX14, resulting in cell proliferation in the cambium. Together with TDIF-PXY signaling, EPFL-ER signaling controls vascular patterning. In addition, the PXY pathway activates BIN2, a GSK3 protein that inhibits the expression of the transcription factor BES1, control xylem differentiation, and cambium activity through phosphorylation of MP/ARF5. Ethylene-induced ERFs, cytokinin-induced ANT, and CYCD3;1 are involved in cambial cell proliferation. The TDIF-PXY pathway also participates in crosstalk with hormonal pathways including auxin, ethylene, and cytokinin. MORE LATERAL GROWTH 1 (MOL1) is another receptor that opposes PXY activity in cambium regulation. In a parallel pathway, the MADS box transcription factors SOC1 and FUL limit cambium activity by repressing AHL15 expression. AHL15, in turn, promotes cell division activity of the cambium by enhancing the expression of cytokinin biosynthesis genes. Xylem formation and auxin maximum in protoxylem cells induce the production of cytokinin through TMO5-LHW pathway. TMO5–LHW directly upregulated LONELY GUY 3 (LOG3) and LOG4 through cytokinin biosynthesis in the xylem axis, cytokinin diffuses to the procambium, where it promotes periclinal cell divisions and stimulates the auxin efflux carrier proteins PIN1 and PIN7, which direct auxin flow toward the protoxylem. Auxin also induces AHP6 expression and suppresses cytokinin signaling in the protoxylem. ACL5 antagonizes TMO5-LHW activity by stimulating SACLs, which can also heterodimerize with LHW.

Various approaches and systems have been used to study the mechanisms governing plant vascular patterning and to identify multiple genetic components and the networks important for vascular patterning and development [15,16,17]. The differentiation of procambium/cambial cells into xylem and phloem is a complex process, and the research on the molecular regulation mechanism of xylem and phloem is also constantly being explored in plants, as shown in Figure 1. As for phloem specification, the most important “conduit” for maintaining the transport of plant nutrients, *SUPPRESSOR OF MAX2 1-LIKE3* (*SMXL3*), *SMXL4*, and *SMXL5* are indispensable regulators of phloem formation. Among these, *SMXL5* facilitated the differentiation of phloem and distal cambium cells and promoted the formation of secondary phloem during the growth of the radial stem [18,19,20]. The Phloem intercalated with xylem (PXY)/Tracheary differentiation inhibiting factor (TDIF) receptor (TDR) involved in the differentiation of xylem cells and proximal cambium cells [19,21,22]. The *ALTERED PHLOEM DEVELOPMENT (APL)* expressed in protophloem sieve elements (PSEs), metaphloem sieve elements (MSEs), associated companion cells (CCs), and phloem-pole pericycle (PPP) cells, which promotes the differentiation of phloem cells [23,24,25]. *CLAVATA3/ESR-like* (*CLE25*), *CLE26,* and *CLE45* genes for the secretory peptides repress PSE formation [26,27,28,29]. Phloem-enriched Dof transcription factors (PEARs) help the phloem formation by activating the precursors of phloem and surrounding cell division [25,30,31,32]. In the mature inflorescence stem of *Arabidopsis thaliana*, fluorescence-activated nuclear sorting (FACS) and laser capture microdissection approaches with next-generation RNA sequencing have provided genome-wide gene expression maps, covering more than 15,000 genes as being differentially expressed among vascular bundles, the proximal and distal cambium, phloem and other stem tissues at different development stages [31]. As for procambium/cambial, PXY [8,17,21], the leucine-rich repeat receptor-like kinase, which is pivotal in cambial cell proliferation, activated by its ligands CLE41 and CLE44, and upregulates the expression of WUSCHEL-related homeobox 4 (WOX4) and WOX14. Another PXY pathway activates a GLYCOGEN SYNTHASE KINASE 3 (GSK3) family protein BRASSINOSTEROID-INSENSITIVE 2 (BIN2), inhibiting the expression of the transcription factor BRI1-EMS SUPPRESSOR1 (BES1), stimulating xylem differentiation. Phytohormones as an irreplaceable factor, ethylene-induced Ethylene response factors (ERFs), cytokinin-induced AINTEGUMENTA (ANT), D-type cyclin CYCD3;1 [33,34], and AT-HOOK MOTIF CONTAINING NUCLEAR LOCALIZED 15 (AHL15) [35] promotes the accumulation of cytokinin and involved in cambial cell proliferation. Auxin, MONOPTEROS (MP)/Auxin response factors (ARFs)-mediated auxin maximum formation and signaling is essential for pro-vascular establishment [36,37], not only in cambial specification but also in xylem formation. In protoxylem cells, auxin directly promotes the production of cytokinin related to the TARGET OF MONOPTEROS 5 (TMO5)- LONESOME HIGHWAY (LHW) pathway [37,38]. Recent studies have established the existence of a negative feedback network in xylem, as LHW heterodimer with either TMO5 or TMO5-LIKE 1 (T5L1) increases the activity of SUPPRESSOR OF ACAULIS51-LIKEs (SACLs) by either directly increasing transcription or via ACAULIS5 (ACL5) thermospermine-mediated translational activation, which in turn, represses the TMO5–LHW interaction [39,40]. Moreover, auxin-induced LHW–T5L1 induces expression of *AHP6* in the protoxylem [41] and *AHP6* inhibits cytokinin signaling in the developing protoxylem and stimulates protoxylem differentiation [42]. Meanwhile, in the TMO5–LHW regulatory pathway in the other direction, TMO5–LHW directly upregulated LONELY GUY 3 (LOG3) and LOG4 through cytokinin biosynthesis in the xylem axis [38].

Undeniably, well-developed vascular bundles can provide strong mechanical support for plants and establish more efficient transport capacity, which can increase the longevity and viability of plants [33], and might have some role in higher yield. There is a more urgent and practical need to extend the study of vascular systems presently, in the context of severe environmental conditions, through different biomolecular strategies to improve crop yield. In this review, we will focus on recent advances in how vascular bundles assist the plant material transport and distribution as a “flow” system, how vascular bundles participate in plant architecture regulation, and how vascular bundles interact with the external environment to improve crop yield, to provide information on appropriate biomolecular methods for obtaining high-yield varieties.

## 2. Advances in Vascular Bundle and “Source-Flow-Sink” Allocation

The biomass production of crops is mainly determined by the accumulation and efficiency of photosynthetic products. However, the economic yield of crops that bear seeds or fruits is influenced by both the efficiency of photosynthetic activity and the translocation of photosynthetic products [43,44,45]. Therefore, while improving the photosynthetic efficiency, it is also important to improve the transport and distribution capacity of photoassimilates. Collaboration of “source-flow-sink” as the main factor affecting photosynthate allocation, sufficient source, smooth flow, and large storage capacity is the ideal breeding target and helps to maximize the transport and distribution capacity of photosynthates to balance source–sink dynamics [46]. Numerous studies on source and sink systems have been conducted that can be seen when we search for critical information, but less progress has been made in crops’ understanding of flow and the vascular bundles (mostly the phloem) systems. [47]. In the studies of vascular bundle development in leaves, researchers only focused on increasing the yield of carbohydrates in leaves in the early years [44,48,49,50,51]. Additionally, they considered the enhancement of the loading capacity of carbohydrates in leaf phloem and the improvement of the unloading and storage of leaf carbohydrates in recent years [52,53,54,55].

In rice, *AtSUC2* (phloem-specific sucrose transporter 2) ectopic expression transgenic plants showed larger grains and more 1000-grain weight than wild types (WT), which increased crop yield by enhancing sucrose loading [56]. Knockout of *OsSUT1* (sucrose transporter) reduced the growth and grain yield in rice [57]. Overexpression of *PbSUT2* from *Pyrus bretschneideri* in tomato (*Solanum lycopersicum* L.) led to increases in the photosynthetic rate in leaves and sucrose content in mature fruit [58], and inhibition of LeSUT1 in tomato leading to transgenic plant phloem loading being blocked and the inability to grow into normal plants [59]. In addition, overexpression of *SUT1* in pea enhanced sucrose allocation to sinks, helping transgenic plants to produce more seed protein and starch, as well as higher seed yield [60]. These results suggested that SUT/SUC-type carriers are mainly responsible for importing sucrose into the phloem sieve element-companion cell (SE–CC) complex, a necessary component part in phloem loading and thus affect “source-flow-sink” allocation to help crop yield improvement.

Another critical sugar transporter, SWEETs (sugars will eventually be transported transporters) [52,61], is also closely associated with crop yield by affecting phloem unloading and “source-flow-sink” allocation. In rice, OsSWEET14 is localized in the vascular parenchyma cells and the nucellar projection, cooperates with OsSWEET11 during the grain-filling stage, and participates in the apoplasmic pathway for sucrose supply to influence the yield [62,63]. In maize (*Zea mays*), ZmSWEET13a, b, c involved in phloem loading, and compared to the WT, the *zmsweet13a, b, c* triple-knockout mutants showed stunted phenotype, with shorter and narrower leaves, which affects yield seriously [64]. Applied to the cultivation and production, Mathan et al. [65] investigated that the vascular development and source–sink relationships can seriously affect the balance between biomass and grain yield in cultivated rice, *Oryza sativa* cv. Nipponbare (optimized for high grain yield), and wild rice, *Oryza australiensis* (high biomass with poor grain yield).

The size, number, and capacity of vascular bundles influence the photoassimilate transportation efficiency. There are 16 traits associated with vascular bundles in different tissues that were investigated in a worldwide collection of 529 *Oryza sativa* accessions, combined with a genome-wide association study (GWAS). Liao et al. [47] screened out 42 and 93 significant association loci in the rice neck panicle and flag leaf and identified *Grain number, plant height, and heading date7* (*Ghd7*) that might strengthen the capacity of transporting assimilates from source to sink, affecting the vascular bundle in the neck panicle at the heading stage. High-throughput SNP markers were used in GWAS to screen 248 maize inbred lines for vascular bundle-related phenotypic loci and identified 15, 13, 2, 1, and 5 SNPs significantly associated with the number of small and large vascular bundles, the average area of single small and single large vascular bundle, and cross-sectional area, respectively [66]. In rice, *OsOPS1* (*OCTOPUS*) was identified in a co-expression network grain size module, its Arabidopsis homologues *OPS* and *OPL2* regulate phloem differentiation and vascular patterning [67,68]. While two mutant lines (*osopl1-1* and *osopl1-2*) showed a significant decrease in grain width and 1000-grain weight compared with WT and displayed other phenotypes, such as reduced plant height, smaller panicle size, and a decreased number of vascular bundles [69] that might participate in the brassinolide pathway. In tomato, Nam et al. [70] found that a reduction in the expression of a negative regulator of phloem development, SlJUL, increases the number of phloem cells and sucrose transport activity and helps in improving 37% of fruit setting and 60% of yield content by suppressing the translation of a positive regulator of phloem development, *SlSMXL5*.

Exploring more quantitative trait loci (QTL) controlling “flow” organs and pleiotropism genes regulating both “flow” and “source–sink” balance to analyze their functions will provide important theoretical and practical significance for polymeric breeding and cultivar improvement.

## 3. Vascular Bundles Development in Establishing Ideal Plant Architecture

In the 1960s, Donald [71] first proposed the concept of designing an optimal plant architecture (ideotype) for wheat. Subsequently, the International Rice Research Institute proposed an “ideal plant architecture (IPA)” characterized by fewer tillers for low yields, more grains per panicle, and thicker stems in the 1990s [72]. With extensive study of various plants, more and more crops have put forward the concept of IPA, such as for rapeseed (*Brassica napus* L.) [73,74], maize [75,76], cotton [77], and other crops. Undoubtedly, they share some desirable plant characteristics that can be used as breeding targets, which are suitable for mechanized cultivation, help plants adapt to survive in different environments, and increase crop yields [78,79]. These studies suggested that vascular bundle development can contribute to the establishment of IPA and increase crop yield.

Plant height is one of the decisive factors for crop architecture, which indirectly affects lodging resistance, plant density, and other characteristics, thereby affecting crop yield [80]. In sesame (*Sesamum indicum* L.), thousand seed weight (TSW) of dwarf mutant *dw607* (*dwf1* type, *SiDWF1* encodes a gibberellin receptor GID1B-like protein) was significantly increased (*p* < 0.01) compared with WT; however, the plant height and internodal length declined by more than 40% and 50%, respectively. Furthermore, cytological analysis of *dw607* (associated with GA throughout plant development) found tightly packed parenchyma tissues around the vascular bundles, which have high lodging resistance [81,82]. Conversely, it has been observed that GA is the specific signal that promotes fibers in the xylem and phloem of cotton plants, which is one of the major goals of the fiber and biomass product industries [83]. The number of xylem fiber cells in the GA 2-oxidase silenced line exceeds that of the GA-20 oxidase overexpressing plant, and its application in the wood and fiber industry can expand production and increase profits [84]. In maize, *GA20-OX1* was highly expressed in the vascular bundle. Overexpression of *GA20-OX1* affects plant height, biomass allocation, and saccharification efficiency [85]. While ectopic overexpression in transgenic Arabidopsis [86] and switchgrass (*Panicum virgatum* L.) [87] of *GA2ox* causes dwarfism and affected stem elongation and lignification. Lodging resistance is highly related to plant height and is also one of the goals of selection and breeding for IPA. In switchgrass, overexpression of *PvWOX3a* promotes cell division, elongation, and vascular bundle development through the increase of GA and cytokinin levels to increase plant height, and surprisedly, *PvWOX3a* and miR156 double overexpression transgenic switchgrass plants show 174% more dry-weight biomass and 162% more solubilized sugars on average than WT [88]. The number of vascular bundles is one of the important factors affecting the mechanical properties of stems [89,90]. Muszynska et al. [91] compared extreme lodging rye mutants and found that the lodging-resistant rye strain “MS135” was taller, with more tiller, had higher biomass production and heavier stalks with significantly more outer vascular bundles, and had a higher proportion of sclerenchyma cells. Recent research has shown that vascular bundles and surrounding leaf sheaths also contribute significantly to lodging resistance [92,93,94,95].

A favorable branch (tiller), as another key factor in IPA, is crucial for biomass production and can help to increase planting density and contribute to a higher yield per unit area [71,96,97,98,99]. Terao et al. [100] identified a vascular bundle systems gene named *ABERRANT PANICLE ORGANIZATION 1* (*APO1*) in rice, which increased the number of large vascular bundles in the peduncle and, consequently, increased the number of primary rachis branches and promoted carbohydrate translocation to panicles. In addition, the *APO1* has been shown to play an important role in the regulation of spikelet number in wheat [101] and inflorescence architecture, floral organ identity, and leaf production rate in rice [102], and is physically associated with LARGE2 in a common pathway to regulate panicle size and grain number [103]. A major rice grain yield QTL *DEP1* increased the number of grains per panicle and caused a consequent increase in grain yield, its NIL-*dep1* plants appeared to have better vascular systems and thicker sclerenchyma cell walls [104]. A study by Jiang et al. [105] overexpressed an OsNAC2 mutant (OerN) and had more vascular bundles (large and small) in stem internodes and leaves. There are two OerN lines that averaged 15.0 and 15.2 primary, and 41.5 and 44.3 secondary branches than parental line averaged 12.2 primary and 31.5 secondary branches. In large-scale field trials, OerN plants produced at least 58.62% more total grain (by weight) compared with the parental line. More in-depth research found that OerN plants showed significant up-regulation of *IPA1* [99] and *DEP1* [104], which are highly associated with grain number and plant architecture.

Leaf angle (LA) as an important character of IPA, is the inclination between the leaf blade and vertical culm [106], the relationship between vascular bundles and LA has also been reported in various studies. Ning et al., [107] reported on a rice mutant, *increased leaf angle1* (*ila1*), which resulted from a T-DNA insertion in a Group C MAPKKK gene, *ILA1*, which is a functional kinase with Ser/Thr kinase activity and predominantly expressed in the vascular bundles. The increased leaf angle in *ila1* is caused by abnormal vascular bundle formation and cell wall composition in the leaf lamina joint. In maize, *SET domain protein 128* (*SDG128*) RNA interference plants show an expanded architecture, less large vascular bundles, more small vascular bundles, and larger spacing of large vascular bundles in the auricles. It has been reported that SDG128 interacts with F-box protein ZmGID2 both in vitro and in vivo, and is involved in maize leaf LA [108]. *ZmSWEET13s*, involved in phloem loading mentioned above, were also significantly associated with middle leaf angle [64].

There are hundreds of biomolecular regulation strategies for IPA, and different characters have different complex regulation mechanisms along with the action of phytohormones, such as auxin [23,74,98], strigolactone [17,23,98], cytokinin [88], and GA [81,82,83,84,85,86,87,88]. Here, we focus on presenting the role and progress of vascular bundles in crop architecture morphogenesis, so other classic plant architecture regulatory networks and strategies will not be elaborated on in detail.

## 4. External Environment Influences Vascular Bundle Development to Affect Crop Yield

Crop yield is a result of quorum sensing. In addition to the coordination and unification of the “source-sink-flow” system of the crop itself, which is one of the important bases of high and stable crop yield, there are external environmental influences such as different cultivation conditions (including increasing planting density, improving the utilization rate of light, temperature, water and fertilizer, and using chemical substances to regulate).

As we mentioned earlier, increasing the planting density contributes to a higher yield per unit area [96], for example, maize production has significantly improved in recent decades due to the optimization of planting density [109,110]. However, with the increase in planting density, the characteristics of the source, flow, sink, and physiological characteristics of crops changed. Based on previous research, Piao et al. [111] used four different cultivation modes to analyze the relationship between increased yield, vascular bundle structure, and post-silking matter transport efficiency of spring maize under high plant density and proved that subsoiling tillage practices and wide-narrow planting patterns could greatly improve grain yield. The application of inorganic fertilizers, to a large extent, helps to improve crop yields, and more importantly improve the utilization efficiency of exogenous nutrients, which is the main goal of breeding [112]. Nitrogen is closely related to the development of vascular bundles, optimized nitrogen fertilizer treatment (OFA) has been shown to enhance the number of branches and spikelets, as well as to improve the diameter and vascular bundle number of panicle-neck internodes [113,114,115]. Researchers have investigated the effects of combining a sensible nitrogen fertilizer selection with greater planting density on grain filling and grain weight. Field experiments prove that planting density and N fertilization level significantly influence vascular bundles in the phloem and xylem, including the total area of the vascular bundles (TAVB) and the proportions of big vascular bundles (PBVB) and small vascular bundles (PSVB) relative to the total vascular bundle area [111], at the same time, the dry matter transport efficiency was also significantly improved. Various studies have proved that when fertilizer is sufficient, source and sink are not the main limiting factor of grain filling rate, but the amount of dry matter accumulation and assimilation distribution to various organs becomes the main limiting factor [116,117]. Future crop productivity will be improved by breeding with a focus on the smooth flow of assimilation distribution to various organs. In addition to inorganic fertilizers like nitrogen, chemicals such as EDAH (a plant growth regulator containing 27% ethephon and 3% DA-6) have been widely used by farmers to increase yield at high densities, which could increase the number and area of stalk vascular bundles [118].

Numerous studies have examined the role of light as an essential growth regulator for plants [119,120], but recently, an interesting study found that light quality plays an important role in watermelon (*Citrullus lanatus*) grafting [121]. The authors describe the quality performance of seedlings under different spectra before and after vascularization and put forward the control mode of light quality, blue light promotes vascular reconnection, while red light boosts the physiological response and quality of grafted watermelon seedlings.

## 5. Conclusions and Future Perspective

**Figure 2:**
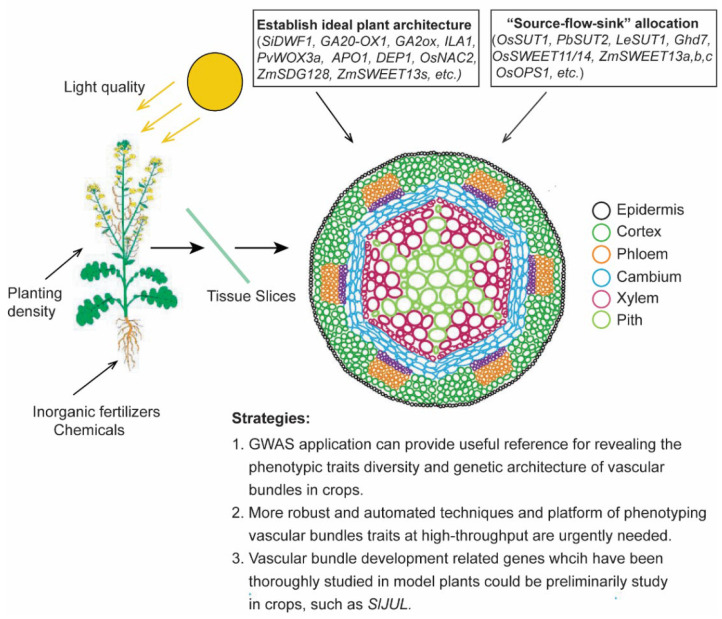
A summary of biomolecular strategies for vascular bundle development to improve crop yield. Environmental factors influence crop vascular system development. And many studies have been carried out on the genes related to vascular bundle systems in crops, which mostly help to establish ideal plant architecture and influence “source-flow-sink” allocation. Several strategies have been proposed here to help explore vascular bundle development.

This review intended to summarize the current research progress of vascular bundles in crops and to emphasize that vascular bundle development has an important impact on crop yield. Compared with the source and sink organs, there is less research on flow organs (vascular bundles), because of the deep location of vascular bundles in the plant body and their complex structure [8,12,13], but the irreplaceability of vascular bundles is undeniable. Global crop production is currently insufficient to meet the anticipated demands of a growing human population. Arable land area reduction and extreme climate change are affecting crop production in many areas, further exacerbating this problem. The demand for increasing crop production in breeding is becoming more and more urgent [1,7] and more attention should be paid to the in-depth excavation of vascular bundles in crops. In this process, we put forward some biomolecular thoughts, which may be helpful for future research on crop vascular bundles (Figure 2).

In this review, we discussed three main topics on how vascular bundle influence “source-flow-sink” allocation helps crop architecture establishment influenced by the external environment. Combined with enhanced crop productivity, as determined by a comprehensive analysis of the biomolecular regulatory networks, it was found that most of the current studies on the development of vascular bundles in crops focus on finding ways to increase the number and size of vascular bundles in order to accelerate material transport and improved the yield. From processed information about vascular bundles in tracheophytes, researchers found that various approaches and platforms have been established to advance our knowledge of the genetic regulation of vasculature development in model plants such as Arabidopsis (Figure 1), from the scale of single genes to networks [14,26,29,30], but these have not been tested in crops. Most of the information regarding the mechanisms underlying vascular bundle growth in plants is still at the level of fundamental understanding and has not been utilized in the advancement of agriculture [8]. Forward genetics is still the most commonly chosen method along with gene discovery by untargeted mutation (or overexpression), followed by the screening of mutants (or transgenics) for a phenotype of interest. The greatest advantage of such screening is its accuracy, but progress is unfortunately slow. As it is difficult to find many relevant mutants in a short period of time, more so for some polyploid crops, for which creating mutants is even harder. At present, many research advances are confined to QTL mapping [47,66]. Further, we found that many vascular systems related to gene mining have also progressed in diploid species, such as rice, maize, tomato, etc. In polyploid species such as rapeseed and cotton, this field is still almost blank. This demonstrates the challenge of studying polyploid species and the fact that there are still plenty of intriguing stories about vascular bundles and yield just waiting to be discovered.

To summarize, an understanding of how vascular bundle development and nutrient transportation increase crop yields is urgently needed in order to meet the challenges of 21st-century agriculture, and there are several biomolecular strategies. First, the construction of a high-quality natural population and GWAS could be precise strategies to capture functional genes and favorable haplotypes for vascular bundle development [47,66]. Second, developing more robust and accurate platforms suitable for detecting anatomical characteristics of vascular bundles is another strategy [122]. With the rapid development of functional genomics and molecular breeding, the ability to quickly screen thousands of lines for targeted phenotypic traits is becoming more and more important. Moreover, vascular tissues are deeply embedded inside the plant body, making phenotypic identification more difficult. Thirdly, some vascular-bundle-development-related genes that have been thoroughly studied in model plants should be preliminarily studied in crops [70]. Blast homologous genes, obtain and check whether transgenic crops can repeat the phenotype to increase yield. Finally, some powerful tools such as bioinformatics can be used to help construct the vascular development genetic network of the same/different species, help to trace the root and analyze the evolutionary relationship, expand our understanding of vascular bundles, and accelerate the research on vascular bundles in improving crop yield.

Vascular bundles, an assembly of (pro)cambial cell files, phloem, and xylem, like a bridge linking and supporting the whole plant body, like a city’s plumbing system, affecting the development, convenience, and safety of a whole plant “city”. Vascular bundles also act as a defense guard, and recent independent studies have reported an innovative vascular-specific immune mechanism to improve the disease resistance of crops to vascular pathogens [123]. It is desirable to understand how to use sophisticated and advanced genetic engineering technology to improve the vascular system of crops, helping them balance the source–sink relationship, enhancing their transport capacity, and achieving the important goal of increasing crop yield from multiple dimensions in limited land and harsh environments.

## Data Availability

Not applicable.

## Abbreviations

ACL, ACAULIS; AHP, Arabidopsis histidine phosphotransfer protein; ANT, AINTEGUMENTA; ARF, Auxin response factor; BES, BRI1-EMS SUPPRESSOR; CLE, CLAVATA3/ESR-like; EPFL, Epidermal patterning factor-like; ER, Erecta; ERL, Erecta-like; GSK, Glycogen synthase kinase; LHW, LONESOME HIGHWAY; LOG, LONELY GUY; MOL1, MORE LATERAL GROWTH 1; MP, MONOPTEROS; PIN, PIN-FORMED; PXY, Phloem intercalated with xylem; MONOPTEROS5TDIF, Tracheary element differentiation inhibitory factor; TMO5, TARGET OF; WOX, WUSCHEL-related homeobox; CYCD3;1, CYCLIN D3;1; AHL, AT-HOOK MOTIF CONTAINING NUCLEAR LOCALIZED; SACLs, SUPPRESSOR OF ACAULIS51-LIKEs; GA, gibberellins; PSEs, protophloem sieve elements; MSEs, metaphloem sieve elements; CCs, associated companion cells; PPP, phloem-pole pericycle; TFs, transcription factors; TSW, thousand seed weight; IPA, ideal plant architecture; QTL, quantitative trait loci; SE–CC, phloem sieve element-companion cell; WT, wild types; FACS, fluorescence-activated nuclear sorting. OFA, optimized nitrogen fertilizer application; TAVB, total area of the vascular bundles; PBVB, proportions of big vascular bundles; PSVB, proportions of small vascular bundles.
